# Implementation of Digital Monitoring Services During the COVID-19 Pandemic for Patients With Chronic Diseases: Design Science Approach

**DOI:** 10.2196/24181

**Published:** 2021-08-26

**Authors:** Luís Velez Lapão, Mariana Peyroteo, Melanie Maia, Jorge Seixas, João Gregório, Miguel Mira da Silva, Bruno Heleno, Jorge César Correia

**Affiliations:** 1 Global Health and Tropical Medicine Instituto de Higiene e Medicina Tropical Universidade Nova de Lisboa Lisbon Portugal; 2 Comprehensive Health Research Center NOVA Medical School Universidade Nova de Lisboa Lisbon Portugal; 3 Research and Development Unit in Mechanical and Industrial Engineering (UNIDEMI) NOVA School of Science and Technology Universidade Nova de Lisboa Caparica Portugal; 4 NOVA School of Science and Technology Universidade Nova de Lisboa Caparica Portugal; 5 NOVA School of Social Sciences and Humanities Universidade Nova de Lisboa Lisbon Portugal; 6 Research Center for Biosciences and Health Technologies Universidade Lusófona de Humanidades e Tecnologias Lisbon Portugal; 7 Instituto Superior Técnico Universidade de Lisboa Lisbon Portugal; 8 Unit of Patient Education, Division of Endocrinology, Diabetology, Nutrition and Patient Education Department of Medicine Geneva University Hospitals and University of Geneva Geneva Switzerland

**Keywords:** primary healthcare, information systems, telemedicine, implementation, design science research, COVID-19, monitoring, chronic disease, elderly, digital health

## Abstract

**Background:**

The COVID-19 pandemic is straining health systems and disrupting the delivery of health care services, in particular, for older adults and people with chronic conditions, who are particularly vulnerable to COVID-19 infection.

**Objective:**

The aim of this project was to support primary health care provision with a digital health platform that will allow primary care physicians and nurses to remotely manage the care of patients with chronic diseases or COVID-19 infections.

**Methods:**

For the rapid design and implementation of a digital platform to support primary health care services, we followed the Design Science implementation framework: (1) problem identification and motivation, (2) definition of the objectives aligned with goal-oriented care, (3) artefact design and development based on Scrum, (4) solution demonstration, (5) evaluation, and (6) communication.

**Results:**

The digital platform was developed for the specific objectives of the project and successfully piloted in 3 primary health care centers in the Lisbon Health Region. Health professionals (n=53) were able to remotely manage their first patients safely and thoroughly, with high degrees of satisfaction.

**Conclusions:**

Although still in the first steps of implementation, its positive uptake, by both health care providers and patients, is a promising result. There were several limitations including the low number of participating health care units. Further research is planned to deploy the platform to many more primary health care centers and evaluate the impact on patient’s health related outcomes.

## Introduction

Since the first case of COVID-19 was diagnosed in Wuhan, China in October 2019, the virus quickly spread around the world to become a global pandemic and a public health emergency, as declared by the World Health Organization in March 2020 [1]. According to the latest available data, more than 180 countries have been affected, with over 178 million confirmed cases including more than 3 million deaths due to the virus [2].

Portugal has also been severely affected by this pandemic, with 420,629 confirmed COVID-19 infections and 6972 deaths by the end of 2020 [3,4]; however, thanks to several important public health measures (eg, closing schools, obligatory used of masks, broad application of testing, and closing of nonessential services) that were quickly put into place by health authorities, the situation was contained, and Portugal has been identified as a moderate success case in managing the pandemic [3]. The Portuguese health system benefits from its universal coverage (eg, all populations have free access to public health, primary, and hospital care in the national health service) and several public health units, which are strategically spread around the country, allowing easy access to care and facilitating contact tracing. Nevertheless, the transmission of the virus persisted and there was a significant increase in mortality rates, particularly among the population over 65 years of age [4] before the start of the vaccination campaign.

It has been observed that older adults and those with underlying health conditions are at increased risk of contracting COVID-19 and have an increasingly rapid and severe progression, often leading to death [5]. Moreover, according to a recent modeling study [6], approximately 1 in 5 individuals worldwide fall into this increased risk category. The increased mortality rate in these populations is expected to be reduced as the vaccination is rolled out, which began in December 2020 [7].

Another cause for concern associated with this pandemic is the severe and widespread disruption of prevention and treatment services for populations affected by chronic conditions. This was reflected in a survey conducted by the World Health Organization [8], involving 155 countries, in which shortages of services for treatment of hypertension (53%), cardiovascular emergencies (31%), for treatment of diabetes and diabetes-related complications (49%), and for cancer treatment (42%) were reported, due to COVID-19 primary health care services disruption. In Portugal, it is estimated that more than 200,000 surgeries and 2 million consultations were delayed due to the COVID-19 pandemic [3].

Several studies [9,10] have also identified the issue wherein people have delayed or avoided seeking medical care for life-threatening conditions, due to concerns about being exposed to COVID-19 in the hospital. Other indirect effects related to the pandemic that have negatively impacted population health were identified, such as an increase in domestic violence and psychological distress related to social isolation [11].

All these issues have led to a renewed focus on telehealth, as a means of providing care to both patients with COVID-19 infections and those requiring other routine clinical services without increasing the risk of potential exposure for patients, clinicians, and staff [12,13].

In this context, we sought to design a web-based digital platform to support primary health care services during the COVID-19 pandemic, by facilitating web-based consultations between primary health care teams and their patients, to guarantee appropriate care, promote adherence to treatment, provide counseling and psychological support.

We aim to share our efforts and experiences in creating a digital health service during a public health emergency (ie, in the midst of a pandemic). This project resulted from a collaboration between academia and health professionals and allowed for the timely deployment (within 3 months) of an innovative digital platform to improved access to health care during lockdown.

## Methods

### Research Method

#### Overview

We designed, created, and implemented a digital platform with specific features in the shortest possible time under difficult circumstances imposed by the pandemic situation using the pace established by Scrum and in a close collaboration with the Primary Health Care professionals who would be using the platform.

**Figure 1:**
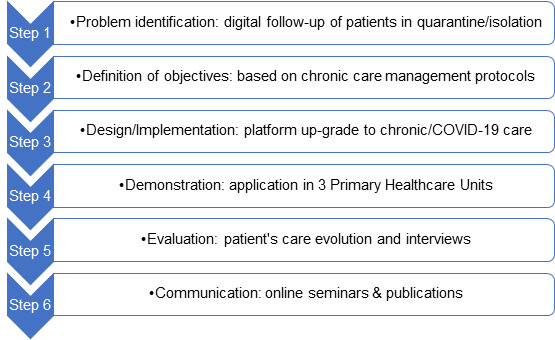
Design science research process.

We followed the Design Science Research Methodology [14,15], which is based on a process with 6 sequential steps (Figure 1). Design Science Research Methodology benefits from supporting the design of an artefact (the primary health care digital platform) to solve the identified problem of lacking the access to chronic patients.

Each step—problem definition, defining the objectives, and design and development, demonstration, evaluation and communication—was performed as a research task.

#### Problem Definition

A set of interviews with general practitioners, nurses, and clinical secretaries helped identify both the critical chronic care processes (eg, consultations frequency and service levels depending on health risks) and the major communication problems between chronic patients and health care professionals (eg, the role of each health professional, how to clearly communicate therapies, and how to improve adhesion), and moreover, in the pandemic, how to access to chronic patients at home.

#### Defining the Objectives

The aim of this activity was to identify the digital care services required by the patients when they interact with their primary health care providers, specifically during lockdown due to the pandemic. This helped to inform the functionalities that the health professionals felt were lacking in care, supported by functionalities that were intended to mimic the work processes of the goal-oriented care methodology for primary health care provision [16]. Goal-oriented care is a patient communication strategy that aims to increase patient responsibility by engaging and involving them more in their therapeutic decision-making process.

#### Design and Development

The design and development of the digital platform was based on the goal-oriented care methodology for primary health care provision [16] and on an existing instantiation that provides digital pharmaceutical services by interacting with chronic patients at home [15]. The use of goal-oriented care principles (ie, creating mechanisms for sharing decision making with patients) allowed us to design service characteristics and functionalities specifically required by chronic patients (eg, the possibility of teleconsultations that enable more frequent consultations as demanded by the patients’ needs and choice of monitoring indicators for more comprehensive evaluation of the patient)). This task was based on Scrum [17] using 1-week sprints with Trello [18]. Scrum is an agile project management method that collects feedback from end-users at the end of each sprint, and new features are prioritized often to reduce risk and extract maximum value [17]. Furthermore, in this project, after the end of each sprint, a new working version of the platform was published and immediately tested by the general practitioners.

#### Demonstration

The platform was then applied in practice engaging the 3 family health units’ professionals for 4 weeks. This allowed us to test online medical and nursing teleconsultations as well as disease monitoring through patient-shared health data.

#### Evaluation

Data from the demonstration were collected. The platform utility was then evaluated based on the teleconsultations effectiveness and patient’s adherence to care, as well as major bottlenecks in users’ experiences. The evaluation also provided more information about the usefulness of the digital service and identified missing features for implementation in the next design cycle. This step will be fully completed after 12 months of the deployment of the platform.

#### Communication

This activity aims to disseminate the project’s results. This activity will be performed throughout the duration of the research project. It includes webinars, oral communications, papers published in conference proceedings within the fields of study and papers to be published in peer reviewed journals.

The project received Ethics approval from the Lisbon Health Region Ethics Commission, and in accordance with international security (ISO 27001) and privacy (General Data Protection Regulation) standards.

### Health Care Setting

In Portugal, primary health care is largely provided by a publicly funded National Health Service System. Regional Health Authorities ensure access through primary care through primary care trusts. These trusts coordinate different types of primary care practices (family health units, public health services, home care services, and allied health professional services) and support units (the executive director, a clinical council, and a management unit) to around 150,000 to 400,000 citizens. Primary care is provided by smaller practices (family health units), generally composed of teams of 15 to 30 health care providers, with 6 to 11 family physicians, an equal number of family nurses, and a smaller number of administrative staff and trainees. These practices serve a fixed list of around 15,000 citizens [12].

There is a mandatory use of electronic health records and electronic prescription; however, the use of videoconferencing is not widespread, mostly because health care units do not have the proper technical equipment for teleconsultations (eg, no cameras are available).

The primary health care digital platform was piloted during the month of July 2020, in 3 family health units (USF for *Unidade de Saúde Familiar* in Portuguese) in the Lisbon Health Region. A USF is an operating unit of primary health care centers with functional and technical autonomy, where patients are closely managed and monitored by a group of primary health care professionals, thus representing an ideal site for our pilot project.

The pilot project included 3 USFs that were purposely selected. *USF Jardim dos Plátanos* is a model A family health unit (those under a commissioning agreement for services delivered), located in a suburban area of Greater Lisbon. At the end of 2019, there were 15,011 registered users and the team consisted of 20 professionals: 8 doctors, 7 nurses, and 5 clinical secretaries. The USF also included family medicine residents and assistant nurses and specialists, as well as technical assistants. *USF das Conchas* is also a model A family health unit, located in Lisbon. There were 13,750 registered patients, 8 doctors, 7 nurses, and 5 clinical secretaries. *USF Ribeirinha* is a model A family health unit, located south of Lisbon, on the south bank of river Tagus. There were 14,962 registered patients, 9 physicians (and 10 residents), 8 nurses, and 6 clinical secretaries.

These health care units were selected for their previous participation in innovative projects addressing the organization of primary health care digital services and their strategic location in a region with a high population density and with a high number of COVID-19 infections and because of the high prevalence of chronic conditions in registered patients.

### Participants

The new digital service was co-designed (eg, functionalities and design options) involving patients and health care providers.

#### Patients

This research included populations that were vulnerable to the COVID-19 infection, namely older adults and those affected by chronic conditions as recommended by health authorities [19-21]. Participants were recruited by physicians if they were older than 60 years of age or had chronic health conditions such as chronic obstructive pulmonary disease, asthma, chronic heart failure, chronic ischemic heart disease, chronic cerebrovascular disease, diabetes, cancer, or rheumatic conditions under disease modifying drugs. For this initial pilot study, patients with low technological literacy or who did not give their consent were excluded. Patients were contacted by the physicians and provided an email, which allowed them to receive the link and the password to access the digital portal. They were also given an email address to contact the research team if necessary.

#### Health Care Providers

We included physicians and nurses in these 3 practices who were willing to participate in the study. All the teams were led by their USF coordinator. Regular team work meetings (eg, each fortnight or each month) were organized with providers to explain and test the digital platform and, in which, we received their feedback and suggestions.

### Digital Health Service Platform

The digital platform was developed based on an existing system from previous eHealth research projects conducted by members of the research team, namely ePharmaCare [15] and HAITool [22]. The original ePharmaCare platform included the following main functionalities: a record of patients’ diseases and medicines, the user messaging inbox and the possibility of chatting between patients and health professionals, records of patients’ physiological and biochemical data (with graphics), a tool for monitoring mental health and quality of life, and the possibility of pharmacists issuing a monthly report to send to the general practitioner. For each type of user profile—professional or patient—there was a home dashboard set up with the main features for its use, with direct links to the information they needed most.

Design Science Research Methodology was used to adapt the platform to the specific objective of the project—namely to allow primary health care physicians and nurses to monitor patients with chronic diseases and those with COVID-19 infections under quarantine [15-23]—by the research team under a participatory process that involved researchers, digital health experts, primary health care professionals, and patients. The health care team of each participating site worked in close collaboration with the researchers (monthly meetings were scheduled for analyzing the advances and collecting suggestions to improve the platform), in order to ensure that the platform responded to their needs (thus guaranteeing adoption and sustainability).

In terms of architecture, the primary health care digital platform (METHIS: Multimorbidity Management Health Information System) is a web-based app with 3 relational databases using PostgreSQL. There is a staging database to enable proper testing before production, a production database to collect USF data, and a research database, with anonymized data from the production database. The platform was also integrated with Zoom (Zoom Inc) to allow for teleconsultations.

We expected that the digital platform would work as a chronic disease management portal, where health care professionals, patients, and caretakers could manage patients’ chronic conditions. It was built to allow both synchronous (consultation videoconferences) and asynchronous communication (patients can record health goals, vital signs, condition-specific scales or general health-related quality of life instruments, and results of investigations).

**Figure 2:**
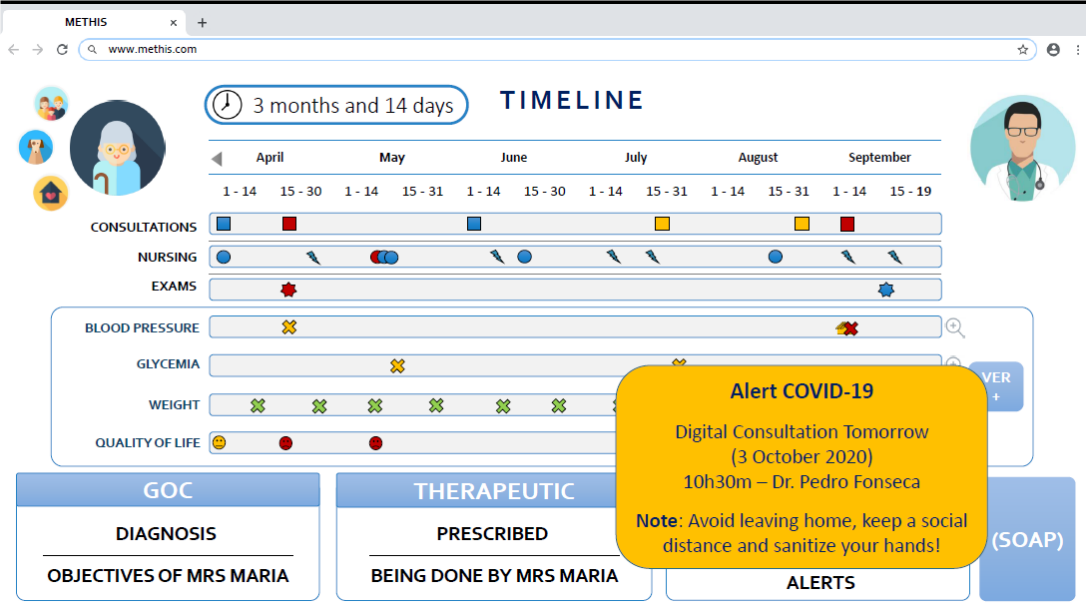
Web-based user interface of the platform (example from a fictional case).

The digital platform promotes and facilitates communication, by including integrated views of the patient’s demographic information, chronic conditions, medication, exams, and personal goals—inspired by goal-oriented care [16] and displayed with innovative layouts and graphics (Figure 2).

The platform presents a set of functionalities that allow scheduling and management of consultations using telephone (voice call) or via digital videoconferencing using Zoom. Teleconsultation scheduling requires only 3 clicks (to select the date, hour, and confirm the consultation that sends the alert to the patient). The platform is also a decision-support system, displaying alerts if patient inputs are out of the specified range upon automonitoring (eg, blood pressure measurement or blood glucose levels) and for drug interactions. The platform also manages and informs patients regarding COVID-19 infection and safety measures. All patient-related health information is processed in a secure manner in accordance with General Data Protection Regulation rules.

### Research Team

The project was proposed and implemented by a multidisciplinary team of 9 researchers, mainly from *Universidade Nova de Lisboa* (with the coordination), including also researchers from 3 other institutions, namely *Instituto Superior Técnico* and *Universidade Lusófona* in Portugal and the Geneva University Hospitals in Switzerland.

The research team comprised 3 physicians (professor of general practice and general practitioner at one of the participating practices, professor of global health and tropical medicine, and senior researcher in chronic disease management and therapeutic patient education), 1 nurse and PhD student, 1 professor of community pharmacy, 1 expert in health communication and PhD student, 1 professor of information systems, 1 expert in digital platforms and PhD student, and 1 professor of health information systems as the team leader (and leader of ePharmaCare [15]).

Part of the team was focused on the development of the digital platform and on direct contact with the family health units (tests and discussions), while the other part of the team provided problem-solving expertise (more often during Scrum meetings but also when specific problems required additional contact).

## Results

### Overview

The project started in May 2020 and by the beginning of July 2020 the platform had been fully deployed (within 9 weeks) and delivered to the 3 family health unit coordinators. We provided 2 tablets (with cameras for teleconsultations) to each USF. A kick-off session was organized with each USF to facilitate the adoption of the platform and address any questions. Every USF performed their first consultation with a real patient during this meeting (the patients were contacted beforehand).

The research problem had been previously defined and the project had identified that there were barriers to communication with chronic patients, such as patients’ lack of mobility and lack of information between the consultations [15].

To define and validate the objectives of a solution (eg, what were the functionalities to be implemented in the digital portal to help link health professionals with patients), focus groups in each of the 3 USFs were organized, and a set of functionalities were specified for integration into the digital platform. All the functionalities that were proposed were validated in the Scrum meetings.

The Scrum meetings took place every week (Monday morning, 60-minute maximum duration) and were supported by Trello to manage the information about the sprints. The meetings started with analyzing the previous week’s sprint, validating the activities that were accomplished as planned, discussing feedback from end-users, and defining a new delivery date for the activities that were, for any reason, not finished as expected. In the second part of the meeting, a new sprint that took into the project timetable into consideration, including who was responsible for each activity, was defined.

Next, the process of designing the new digital platform was developed in collaboration with the physicians, nurses, and clinical secretaries of the 3 USFs. Often the physicians of our team would help clarify requirements for a functionality, and the digital platform team would analyze how it could be implemented. In every Scrum meeting, validated functionalities were assigned to a timeframe, to be developed and integrated into the platform. Nonvalidated functionalities were transferred to staging, for future consideration. After the first set of meetings, it was decided that the platform would include 5 priority features.

### Features

#### Teleconsultations

This feature allows appointment to be easily booked for teleconsultations. Prior to the appointment, a message is sent to both physicians and patients with the schedule and the unique link to the teleconsultation. It also included a SOAP (Subjective, Objective, Assessment, Plan) free-text structure, as demanded in primary health care settings [24]. If part of this information was of interest to the patient, it could be also shared with them, with a simple click to validate this option.

#### Monitoring Patient Data

This feature allows monitoring of patients’ biochemical and physiologic data as well as prescribed medicines, with several graphic displays available to facilitate interpretation and follow-up. This data can be uploaded by the patient (at home or after examination at the community pharmacy) or by the physician (at the consultation).

#### Patient Participation

This feature allows patients to upload daily or regular physiologic and biochemical data that can be later visualized by physicians or the patients themselves. Patients can also ask questions and share information with the health professionals via a chat system, in addition to receiving information sent by the health professionals.

#### Alerts

The platform has new smart components, including algorithms that allow a set of alerts to improve physicians’ and nurses’ responses to a potential negative evolution in patients’ data. As an example, all physiological and biochemical indicators have healthy limits included in the system, and whenever the indicator is outside these limits, or whenever this indicator is n-times outside the predetermined normal interval defined by the physician, it issues an alert.

#### Medicine Management

Using the Portuguese medicine database webservices provided by the National Authority for Medicines and Health Products (INFARMED), we included a module for therapeutic management. This module allows professionals or patients, to input the name of their medication (generic or commercial name) and dosage to yield a therapeutic profile. A feature of this profile is to automatically calculate the end date for each medication package prescribed, assisting with medicine management and therapeutic adherence issues. This feature, alongside the participation of a specialist pharmacist, will allow for different types of medication reviews and enhanced pharmaceutical care. This last function was inherited from the ePharmaCare project.

### Demonstration and Evaluation

The demonstration took place after a meeting with USF teams in July 2020. There was a positive uptake by health care providers and patients in a truly short term. As of December 31, 2020, there were 53 health care providers (37 physicians and 16 nurses) from 3 USFs engaged and using this platform to follow 35 patients (Table 1).

In order to provide the first evaluation of the digital care provided, during the sequential meetings and first tests, we observed, listened, and recorded health care providers talking about their experiences and difficulties while using the platform.

At 3 kick-off meetings with USFs (in the beginning of July), the physicians and nurses showed marked enthusiasm. In all occasions, they immediately took the new tablets with the system and started using them without almost any help from the research team.

Interestingly, in the first presentation of the digital platform by the researchers, during which physicians were told that they could define functionalities that they needed to be added to the platform, 1 physician enquired whether they could really ask for additional functionalities for the system.

We observed that the first patient at USF *Jardim dos Plátanos*, a retired lawyer of 83 years of age (who provided written consent), acknowledged the reception of the email with the log-in and password. Soon after, he managed to initiate the teleconsultation easily, without any help from the physician.

A physician, during her first consultation and while talking with the patient, managed to effortlessly book another appointment (scheduled for after the holidays, 1 month afterwards) and requested that the patient enter his weight and arterial pressure every week in the platform. The patient said he would try.

Several physicians and nurses pointed-out the user-friendliness of the digital platform, and the reduced number of clicks necessary to perform the basic functions (eg, a teleconsultation with a patient requires only 3 clicks including log-in to the platform).

**Table 1:** Description of the number of health care provider and patients per unit until December 31.

USF^a^	Physicians, n	Nurses, n	Patients, n	Teleconsultations, n
A	15	7	11	12
B	13	7	7	8
C	9	2	17	21
Total	37	16	35	41

^a^USF: family health unit (*Unidade de SaúdeFamiliar*).

In 1 USF, the 2 physicians mentioned that the platform could also be used to follow pregnant women, since the protocols are well-known, and it would reduce their COVID-19 infection risk (and they already have used it for that purpose).

It was observed that each USF’s team preferentially used different functionalities of the platform that were in line with the work and organizational processes of each site. Indeed, while 1 USF preferred to use the platform mostly for teleconsultation, another used it more for the recording and monitoring of patient data and another to focus on engaging more digitally literate patients. Understanding this behavior could be a possible focus for a future study.

Several physicians mentioned difficulty getting the tablet (often kept in a lockable drawer) when they were busy with consultations in order to perform teleconsultations. At USF *Ribeirinha*, a clinical secretary was called to help tackle this barrier.

The USF coordinator at *Conchas* was naturally skeptical at the beginning, but soon after using the platform, he accepted the challenge and assumed the leadership of using the system in his USF. In the meantime, we had a meeting at this USF with the National eHealth Institute, where this coordinator played an important role in promoting the value of the platform.

An important measure of some difficulties was the slow rate of adding new patients. This was, the health professionals said, in part, limited by the holiday season and related absences of many health care providers and patients. Logistic difficulties due to COVID-19 were also a barrier.

Important features of the digital platform that had not been possible before were the ability to contact and see the patients (not possible in the telephone calls) and to make appointments with reminders for upcoming teleconsultations. We were told that several patients had already started entering their own data onto the platform.

Regarding dissemination, in addition to an oral communication presented to a special COVID-19 session [25], a press release was also sent to the media that resulted in an interview in the Portuguese Public Television. More importantly, this paper fulfills the methodological aim.

## Discussion

**Figure 3:**
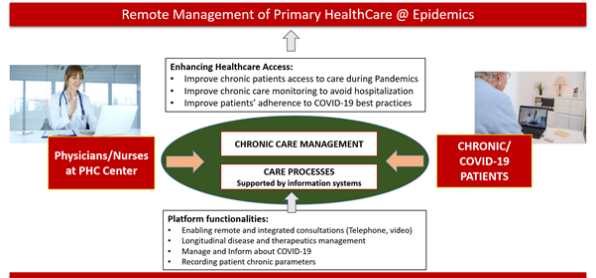
Overview of the project. PHC: primary health care.

The main objectives of this rapid implementation project were attained: a digital platform was designed, implemented, and demonstrated in 3 different USFs, with several health care providers already using it to manage a set of their patients remotely (Figure 3).

The rapid implementation (in 9 weeks and within the 3-month limit, as required by the funding entity) was focused on the development of the platform, with main organizational implications, such as communication with patients and the number of interactions (many now through digital media).

Tablets were chosen for 4 reasons: camera, easy access to the internet, inexpensive, and easy to use. They can be used by different physicians and nurses throughout the day and provided the capabilities for performing both teleconsultations and monitoring. The results thus far have provided valuable proof-of-concept feedback and proved that health care professionals in the USFs could provide remote consultations to their patients during the COVID-19 pandemic using our digital health service.

We observed that health care providers were able to safely communicate with and prescribe appropriate treatment to their patients, as well as inform them about the COVID-19 related protective measures and answer any other queries they might have (eg, mental health). Yet, we did not have the opportunity to assess patients either on quarantine or in isolation as, at the time of implementation, the rate of COVID-19 infected patients was still relatively low in Portugal (approximately 0.8% [26]), and the physicians at the 3 USFs reported 1 or fewer infections in their populations. However, physicians were reportedly very busy taking care of chronic patients, using either telephone calls or emails, as most USF stopped attending patients in person. The competition, usual in any innovative process, between the 2 solutions (telephone vs digital platform) seemed to tend toward the simplest option of the telephone call, as the physicians claimed to be overloaded. Yet, 2 to 3 health professionals from each USF were enthusiastically looking for opportunities for using the digital platform, expecting to obtain the most relevant patient information from their patients with more digital proficiency.

There are similar projects that are currently being developed in countries all over the world, such as in Canada [1,2], the United Kingdom [3], and China [4-6], with promising results that suggest that, if implemented and delivered appropriately, digital health solutions can effectively reduce the burden on hospitals, prevent overcrowding, reduce the risk of cross-infection, and relieve patient anxiety.

Although we have not completed the evaluation of our service, there is increasing evidence that clinical consultations conducted through a video link tend to be associated with high satisfaction among patients and staff, with no difference in disease progression and lower costs compared with traditional clinic-based care [10]. In fact, several recommendations are being developed to help primary health care professionals remotely provide COVID-19–related consultations [11]. One aspect to be taken into consideration is the actual support of the clinical secretary in managing the first level of communication with the patients as envisaged in the beginning of the project (ie, the digital portal includes the role of the clinical secretary), which is a functionality that most physicians did not take into consideration.

Nevertheless, there is still a lack of national and local guidelines regarding the remote management of patients using digital health solutions, and urgent research is required in this field. Our research efforts may contribute, albeit indirectly, to solving this problem. Other risks of using this type of platform include the possibility of misdiagnoses, equipment malfunction, and privacy breaches [8]. Other issues that need to be addressed include inadequate training of health care providers of skills in dealing with remote consultations to provide safe and effective patient care [9]. And, of course, the widespread use of resources, such as those demonstrated herein, is always dependent on patients’ adequate technological literacy and internet access [27].

Other researchers have also highlighted the importance of implementing digital solutions without further fragmenting the existing landscape of care [13]. This requires concerted efforts between policy makers, researchers, and health professionals that take into account feedback from the patients. In Portugal, the primary health care activities are annually commissioned, to target the number of chronic care procedures to be accomplished but not the comprehensive well-being of the patient [12].

This digital platform will also be a valuable source for epidemiological data. We are also currently discussing different functionalities that can be used for research purposes (eg, to evaluate the effectiveness of a digital program for diabetic patient education using a virtual assistant, or the follow-up of disease-specific cohorts). The project team was recently contacted by a set of health care centers inquiring how to be included in the study, and by African Portuguese-speaking country partners, as they have shown interest in this platform for advancing universal coverage in their countries.

Although still in the first steps of project dissemination, we are seeing promising results with a positive acceptance by health care providers and patients. The primary health care units now have a digital platform for following and monitoring chronic patients, which can be use in the eventuality of subsequent waves of COVID-19 infections or during the flu season.

This study had several limitations. Most notably, the results may not be generalizable, as only 3 health units participated in the study, and it was developed under the restrictions of COVID-19 pandemic (eg, several meetings with health professionals were conducted via videoconference). Further research is required to evaluate the impact on patient health-related outcomes. We believe that this platform could be scaled-up to many more primary health care centers in Portugal.

## Abbreviations

USF: family health unit (Unidade de Saúde Familiar)
